# Ultrasound verified inflammation and structural damage in patients with hereditary haemochromatosis-related arthropathy

**DOI:** 10.1186/s13075-017-1448-0

**Published:** 2017-10-24

**Authors:** Christian Dejaco, Andreas Stadlmayr, Christina Duftner, Viktoria Trimmel, Rusmir Husic, Elisabeth Krones, Shahin Zandieh, Emma Husar-Memmer, Gernot Zollner, Josef Hermann, Judith Gretler, Angelika Lackner, Anja Ficjan, Christian Datz, Roland Axman, Jochen Zwerina

**Affiliations:** 1 Department of Rheumatology and Immunology, Medical University Graz, Auenbruggerplatz 15, A-8036 Graz, Austria; 2 Department of Rheumatology, Hospital of Bruneck, Spitalstrasse 11, 39031 Bruneck, Italy; 3 Department of Internal Medicine, General Hospital Oberndorf, Teaching Hospital of the Paracelsus Medical University Salzburg, Paracelsusstraße 37, 5110 Oberndorf bei, Salzburg Austria; 4 Department of Internal Medicine VI, Medical University Innsbruck, Anichstraße 35, 6020 Innsbruck, Austria; 5 Department of Radiology, Medical University Graz, Auenbruggerplatz 9, 8036 Graz, Austria; 6 Department of Gastroenterology and Hepatology, Medical University Graz, Auenbruggerplatz 15, 8036 Graz, Austria; 7 Institute of Radiology and Nuclear Medicine, Hanusch Hospital, Heinrich-Collin-Straße 30, 1140 Vienna, Austria; 8 First Medical Department, Hanusch Hospital and Ludwig Boltzmann Institute of Osteology at the Hanusch Hospital of WGKK and AUVA Trauma Centre Meidling, Heinrich-Collin-Straße 30, 1140 Vienna, Austria

**Keywords:** Hand osteoarthritis, Arthritis, Ultrasonography, Haemochromatosis

## Abstract

**Background:**

Chronic arthropathy occurs in approximately two thirds of patients with hereditary haemochromatosis (HH). The aim was to study inflammatory and structural lesions in patients with HH with (HH-A) and without arthropathy (HH-WA) using ultrasonography.

**Methods:**

This was a cross-sectional study of 26 patients with HH-A, 24 with HH-WA and 37 with hand osteoarthritis (HOA). Clinical examination was performed in 68 joints, and we retrieved data on hand function, pain and global disease activity (all using a visual analogue scale (VAS)), morning stiffness and ferritin levels. Standard x-ray and ultrasound were conducted in 36 joints (hands, hips, knees and ankles), and we graded grey scale synovitis (GSS), power Doppler ultrasound (PD), osteophytes, erosions, tenosynovitis and cartilage damage semi-quantitatively in accordance with prior publications.

**Results:**

Ultrasound revealed a high proportion of inflammatory changes in HH-A; GSS was found in 96.2% and PD signals in 80.8% of patients (median GSS score 9, PD score 2.5). The frequency of these findings was similar in HOA. Inflammation was also common in HH-WA, yielding GSS in 83.3% and PD signals in 50.0% of patients. Cartilage damage was most prominent in HH-A as compared to HH-WA and HOA (median scores 11.0, 2.5 and 2.0, respectively). The prevalence and extent of erosions and osteophytes were similar in all groups. None of the ultrasound scores was associated with pain or function; GSS, PD, osteophyte and cartilage scores correlated with x-ray-verified structural damage.

**Conclusion:**

A high prevalence of ultrasound-verified inflammation and cartilage damage was found in HH-A, and to a lesser extent in HH-WA. These findings were associated with x-ray-verified damage but not with clinical scores of pain and function.

## Background

Hereditary haemochromatosis (HH) is the most frequent autosomal-recessive inherited metabolic disorder with up to 0.5% homozygous mutations in Caucasian populations [1]. A mutation in the *HFE* gene encoding a transmembranous glycoprotein leads to increased duodenal iron resorption despite full iron stores [2]. The iron overload causes a characteristic clinical pattern with liver cirrhosis, hyperpigmentation and diabetes mellitus. Additional manifestations are hypogonadism, congestive heart failure and arthropathy [3]. Diagnosis is usually based on elevated iron stores as measured by serum ferritin levels and genetic testing of the *HFE* gene [3, 4].

Chronic arthropathy occurs in approximately two thirds of patients with HH [5]. In the majority of cases, HH arthropathy (HH-A) manifests as chronic destructive joint disease causing similar complaints to osteoarthritis (OA), whereas ≤ 5% of patients suffer from inflammatory arthritis with a comparable disease course to pseudo-gout [5].

Diagnostic criteria for HH-A have not yet been established. The typical clinical picture is bony swelling and tenderness of the 2^nd^ and 3^rd^ metacarpophalangeal (MCP) joints; however, diagnosis may be difficult in cases of mild symptoms and/or involvement of other (particularly large) joints. Treatment of HH-A is challenging because regular phlebotomies do not lead to improvement in joint pain. Analgesics and/or non-steroidal anti-inflammatory drugs (NSAIDs) are frequently used; however, the clinical efficacy of these drugs is variable and it is unknown whether treatment might modify the course of the disease [6].

Imaging studies have rarely been performed in HH-A so far. Conventional radiography is the gold standard method for the detection of structural changes including joint space narrowing (JSN), osteophytes, calcium pyrophosphate deposition (CPPD) and/or subchondral osteosclerosis [7–9]. Magnetic resonance imaging (MRI) has been applied in a few case series and small studies with mixed results. In a series of three patients with HH with pain and swelling of the ankles, for example, MRI identified advanced degenerative changes without notable inflammation [10]. In another MRI study of patients with haemosiderosis (caused by regular blood transfusions due to beta-thalassemia), synovial inflammation of wrists was reported in 23% of cases [11].

Musculoskeletal ultrasound has not yet been used to systematically study synovial and bony changes in patients with HH-A. The aim of this study was therefore to investigate inflammatory and structural abnormalities in patients with HH-A, HH without arthropathy (HH-WA) and hand OA (HOA) using ultrasonography.

## Methods

This was a cross-sectional study conducted at the Medical University of Graz, the Hanusch Hospital Vienna and the General Hospital Oberndorf (all Austria). Approval by the respective institutional review boards and written informed consent of each patient were obtained.

### Patients

Patients with HH and hand OA (HOA) were recruited from clinical routine practice. HH was defined by a homozygous *C282Y* mutation or a compound mutation of the *C282Y* and *H63D* genes plus serological signs of iron overload at diagnosis: increased transferrin saturation (> 55% in men, > 45% in women) and either provisional (serum ferritin > 300 ng/ml for men and postmenopausal women, > 200 ng/ml for premenopausal women) or overt iron overload (serum ferritin > 1000 ng/ml or hepatic iron overload on biopsy) independent of the presence of clinical symptoms related to iron overload [9].

HH-A was defined as HH plus pain in the hands (VAS > 10 mm and/or ≥ 1 tender joint) plus at least one radiographic change compatible with HH-A on hand x-rays [9, 12]. Two patients with HH who declined to undergo x-rays were classified on a clinical basis only; both patients were considered to have HH-WA.

Patients with HOA were classified using the criteria of the American College of Rheumatology [13]. All patients with HOA had a negative medical and family history for HH and they were tested for the absence of serological signs of iron overload.

### Clinical assessments

All patients underwent a structured history/chart review retrieving demographic data, medical history and current medication. Patients with HH were further questioned for the duration and frequency of phlebotomies, previous joint replacement and HH-related co-morbidities including hepatopathy, hypogonadism, cardiomyopathy, skin changes and diabetes mellitus.

Clinical assessments included the duration of morning stiffness (minutes), number of tender joints (TJ) (68-joint count), swollen joints (SJ) (66-joint count), number of bony swollen joints (assessed at the MCPs, proximal (PIP) and distal interphalangeal joints (DIP), wrists, knees, and ankles), patient’s (PGA) and evaluator’s global assessment of disease activity (EGA), hand function and pain related to hands, hips, knees and ankles. Function, global and pain assessments were all recorded on a 100-mm VAS, with 0 = best, 100 = worst [9, 12].

Blood investigations included erythrocyte sedimentation rate (ESR, measured by the Westergren method) and C-reactive protein (CRP) (measured by nephelometry), blood cell count, liver enzymes, renal functional tests and iron status (serum ferritin, transferrin and transferrin-saturation). The results of the *HFE* gene mutation (*C282Y* and *H63D*) tests were also retrieved in order to classify the patients.

### Ultrasound protocol

Grey scale (GS) and power Doppler (PD) sonography were performed in 36 joints (wrists, MCPs, PIPs, DIPs, hips, knees and ankles) by one of two rheumatologists (CDe and CDu) who were unaware of the clinical findings (but not blinded to diagnosis of HH versus HOA). We used a MyLab Twice ultrasound device (Esaote, Genova, Italy) with two multi-frequence linear transducers (6–18 MHz, small and medium joints; 4–13 MHz, large joints). For GS imaging, parameters were adjusted to maximize the contrast between examined structures. PD settings were standardized accordingly: frequency 9.1 (small joints and entheses) or 6.3 (large joints) MHz, pulse repetition frequency 750 Hz and medium persistence. The PD gain was optimized by increasing the gain until noise appeared and then reducing it just enough to suppress the noise [14].

All ultrasonography comprised longitudinal and transverse scans in accordance with current guidelines and publications [15]. GSS was subjectively graded from 0 to 3 in which 0 represented no GSS, 1 = minimal, 2 = moderate and 3 = extensive GSS as defined in recent publications [14, 16–18]. PD signals in large and small joints were also semi-quantitatively assessed on a scale of 0–3 with 0 = no PD-signal, 1 = up to three single or two confluent vessels, 2 = less than half of the synovia and 3 = half or more of the synovia covered by PD signals [14, 16–18]. GSS and PD were independently graded on each view of each joint (e.g. palmar and dorsal) and the highest value for each joint was counted for the sum score. Tenosynovitis was scored as reported previously [14, 19]: GS tenosynovitis (GS-teno) was identified as hypoechoic or anechoic thickened tissue with or without fluid within the tendon sheath and was semi-quantitatively graded from 0 to 3 at the wrists and ankles, whereas at the level of MCPs, PIPs and DIPs it was recorded as 0 = absent or 1 = present. Tenosynovitis affecting the tendon at the level of phalangeal bones was assigned to the nearer joint (e.g. flexor tenosynovitis at the PIP bone was assigned either to the MCP or the PIP depending on which joint was closer). Tenosynovitis covering > 50% of the area of a phalangeal bone was scored for both related joints. At wrists, the extensor and flexor tendons were independently scored using the following grading: 0 = no tenosynovitis, 1 = tenosynovitis at one, 2 = at two and 3 = at three or more tendon compartments. At the ankles, only the extensor tendons were assessed and graded from 0 to 3, with 0 = no tendon, 1 = one tendon, 2 = two tendons and 3 = all three extensor tendons were involved. PD signals related to tenosynovitis (PD-teno) were graded from 0 to 3 (0 = no PD signal, 1 = up to three single or two confluent vessels, 2 = less than half of the tendon/tenosynovia and 3 = half or more of the area covered by PD signals). At the wrists and ankles the tendon with the maximal PD score was counted.

Erosions (assessed at MCPs, PIPs and DIPs only) or osteophytes (assessed at MCPs, PIPs, DIPs, hips and knees) were defined by a step-down or step-up contour defect, respectively that is visible in 2 perpendicular planes [20]. At the MCPs, PIPs and DIPs, erosions and osteophytes were independently assessed on dorsal and palmar views; lesions visible in the lateral compartments (e.g. erosions at the lateral aspects of the 2^nd^ or 5^th^ MCP joints) were counted as dorsal lesions. Osteophytes were further investigated by anterior scans of the hips and at the medial and lateral femorotibial spaces at the knees [15]. Grading of erosions was conducted from 0 to 3 as described previously [14] and was based on the maximum diameter of the cortical break for bone erosions (adapted from [21]) with grade 0 = no erosion, grade 1 = erosion ≤ 1 mm, grade 2 = erosion > 1 mm and ≤ 2 mm, grade 3 = erosion > 2 mm and/or large destruction of the joint. In the case of multiple erosions the largest lesion was counted. For osteophytes the maximum distance between the “original” and new cortical lining (= maximal height) was measured: grade 0 = no osteophyte, grade 1 = osteophyte ≤ 1 mm, grade 2 = osteophyte > 1 mm and ≤ 2 mm, grade 3 = osteophyte > 2 mm and/or large and diffuse osteophytes (adapted from [21]). Erosions and osteophytes were independently graded on each view of each joint and the highest value was counted for the sum score.

Cartilage was assessed at MCPs 2–5 on longitudinal scans with full flexion of the fingers, and at the knees on transverse scans with full flexion of the knees. Scoring was adapted from Filippucci et al. with a grading from 0 to 4, with 0 = no evidence of cartilage abnormalities, 1 = loss of sharpness of the superficial margin of the hyaline cartilage, 2 = partial thickness defect of the cartilage layer, 3 = full thickness defect of the cartilage layer with a normal subchondral bone profile and 4 = complete loss of the cartilage layer and subchondral bone involvement. CPPD deposits were defined as previously described and graded as 0 = absent or 1 = present [22]. Sum scores were calculated for GSS (range 0–108), PD (0 − 108), osteophytes (0–96), erosions (0–84), GS-teno (0–40), PD-teno (0–96) and cartilage (0–40).

### X-rays

Patients with HH underwent standard radiography of the hand and wrist, knee and ankle joints within 2 weeks of the study visit. All radiographs were assessed by one of two experienced radiologists (VT or SZ) and scored from 0 (no damage) to 6 (extensive damage) per joint as previously described [9]. The total score ranged from 0 to 72.

### Statistical analysis

Statistical analysis was performed using SPSS (version 23.0). Descriptive statistics were used to summarize the data. The distribution of data was tested for normality using the Kolmogorov-Smirnov test. For continuous non-parametric data, we show the median and range whereas for parametric data, the mean and standard deviation are depicted. Comparisons between independent groups were conducted using the Mann-Whitney *U* test. Paired categorical data were analysed using the chi square test or Fisher’s exact test as appropriate. *P* values were not corrected for multiple testing.

Inter-rater reliability of the ultrasound results was determined by serial blinded assessments of 10% of patients’ scans by two investigators (CDe and CDu) and using the intra-class correlation coefficient (ICC).

## Results

### Clinical characteristics

There were 24 patients (27.6%) classified as HH-WA, 26 (29.9%) as HH-A and 37 (42.5%) as HOA. Clinical characteristics are detailed in Table 1.

**Table 1:** Patients’ characteristics

	HH-WA (*n* = 24)	HH-A (*n* = 26)	HOA (*n* = 37)
Age^a^ (years)	57.1 (13.0)	57.8 (9.7)	60.1 (9.5)
Female, *n* (%)	8 (33.3)*	5 (19.2)*	34 (91.9)
Disease duration since diagnosis^b^ (years)	7.5 (3.2–22.8)*	10.3 (0.8–27.6)*	0.9 (0–23.5)
Organ involvement HH, *n* (%)			
Aminotransferase elevation	4 (16.7)	2 (7.7)	-
Liver fibrosis/cirrhosis	0	5 (19.2)^¥^	
Impaired glucose tolerance	1 (4.2)	2 (7.7)	
Diabetes mellitus	2 (8.3)	3 (11.5)	
Cardiomyopathy	0	0	
Morning stiffness^b^ (minutes)	0 (0–10)	3 (0–180)	7.5 (0–120)
EGA^cb^ (mm)	0 (0–29)	13.5 (0–61)	10 (1–53)
PGA^cb^ (mm)	5 (0–65)	25 (0–74)	25 (0–77)
Pain hands^cb^ (mm)	0.5 (0–18)	31 (0–72)	24.5 (0–86)
Pain hip^cb^ (mm)	1 (0–44)	10.5 (0–58)	6 (0–91)
Pain knee^cb^ (mm)	0.5 (0–50)	25 (0–76)	10 (0–86)
Pain ankle^cb^ (mm)	1 (0–60)	35.5 (0–96)*	3 (0–86)
Hand function^cb^ (mm)	4.5 (0–66)	39.5 (0–100)	47 (0–92)
Bony swollen joints^b^	0 (0–12)	2.5 (0–14)*	7 (0–24)
≥1 Bony swollen joint, *n* (%)	8 (33.3)	16 (61.5)**	36 (97.3)
Tender joints^b^	0 (0–31)	4 (0–29)	3 (0–40)
≥1 Tender joint, *n* (%)	6 (25.0)	22 (84.6)	30 (81.1)
Swollen joints^b^	0 (0–1)	0 (0–7)	0 (0–6)
≥1 Swollen joint, n (%)	2 (8.3)	6 (23.1)	8 (21.6)
ESR^b^ (mm/1^st^ hour)	9 (0–29)	8.5 (1–36)	6 (2–34)
CRP^b^ (mg/L)	0.5 (0–4)*	0.5 (0–4.2)*	1.3 (0.1–12.6)
Ferritin^b^ (at visit) (ng/ml)	83.6 (28–1060)	66.1 (14–853)	100.0 (19–266)

Pain scores, global assessment, function, morning stiffness, bony swollen joints, tender joints and swollen joints were not compared between HH-WA and HH-A or HH-WA and HOA because by definition, there were differences between these groups

*HH* hereditary haemochromatosis, *CRP* C-reactive protein (normal value 0–5 mg/L), *EGA* evaluator global assessment, *ESR* erythrocyte sedimentation rate (normal range 0–10 mm/1^st^ hour), *HH-A* hereditary haemochromatosis with arthropathy, *HH-WA* hereditary haemochromatosis without arthropathy, *HOA* hand osteoarthritis, *n* number, *PGA* patient global assessment

^a^Mean (standard deviation)

^b^Median (range)

^c^All measured on a visual analogue scale with a range 0–100 mm, with 0 = best, 100 = worst

*p < 0.01, **p < 0.001 compared to HOA; ^¥^ *p* ≤ 0.05 compared to HH-WA; *p* values are not adjusted for multiple testing

As expected, there were more women in the HOA than in the HH groups, and disease duration was longer in patients with HH than in patients with HOA because most patients with HOA were diagnosed at the time of inclusion into the study. Patients with HH-A more commonly had liver fibrosis/cirrhosis than patients with HH-WA, whereas disease duration, ferritin levels at the time of the clinical visit and median duration (6 (0–26) vs. 6 (0–23) years, respectively) and the number of phlebotomies per year (2.5 (0–12) vs. 3 (0–5), respectively) were similar in the HH-A and HH-WA groups. Clinical parameters of joint inflammation and function where higher in HH-A than in HH-WA by definition (see Table 1). Joint replacement was more common in HH-A than in HH-WA (hip, 8 (32.9%) vs. 2 (8.3%) patients, *p* = 0.047; knee, 2 (8.0%) vs. 1 (4.3%) patient, *p* > 0.2). Patients with HH-A had higher levels of ankle pain as compared to patients with HOA, whereas the number of bony swollen joints and CRP levels were higher in patients with HOA than in patients with HH-A.

### Ultrasound findings in patients with hereditary haemochromatosis

**Fig. 1:**
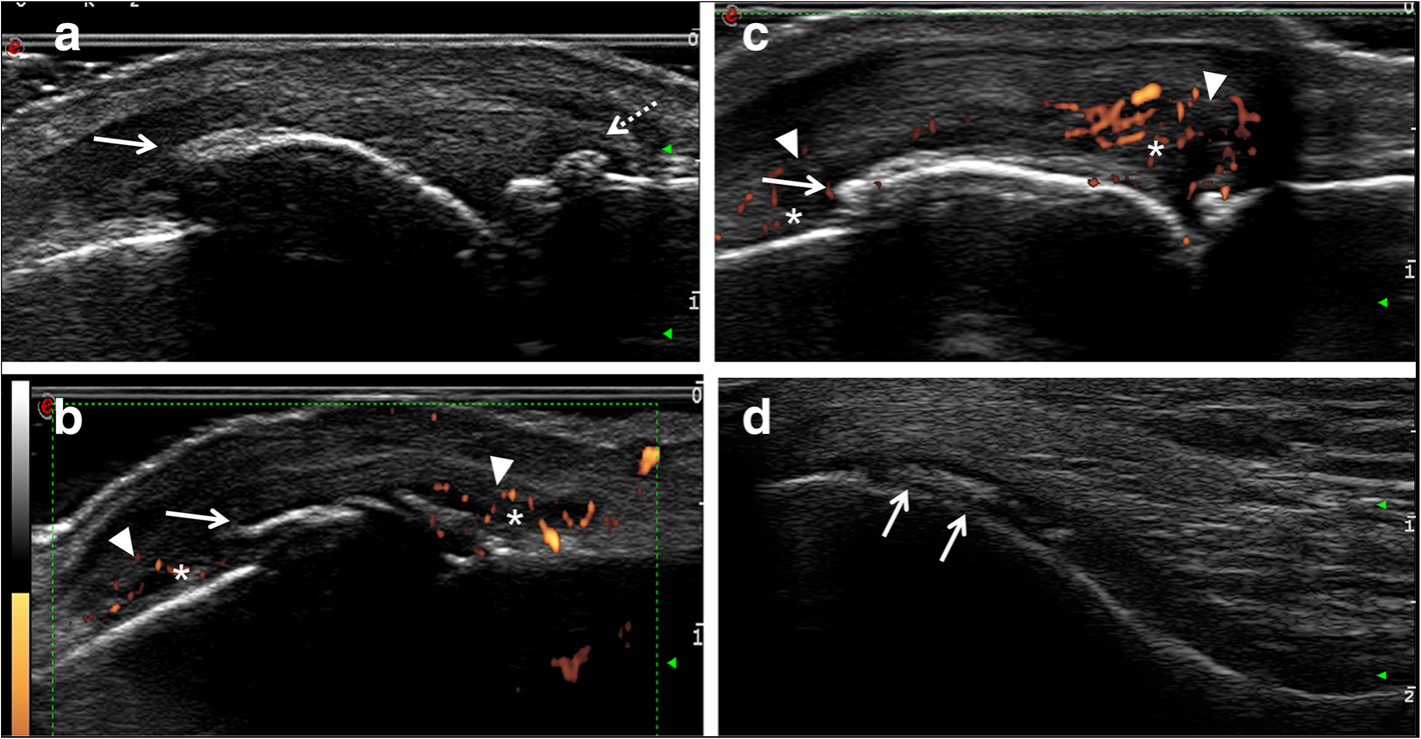
Ultrasound image examples of patients with hereditary haemochromatosis. **a** Longitudinal dorsal scan of a metacarpophalangeal (MCP) joint (left = proximal) indicating an extensive proximal osteophyte (arrow) and a smaller osteophyte in the distal part of the joint (broken arrow). **b**, **c** Longitudinal dorsal scan of MCP joints indicating a proximal osteophyte (arrow), grey scale synovitis (arrow heads) and power Doppler signals (asterix). **d** Transverse suprapatellar scan of a knee indicating calcium pyrophosphate deposition in the hyaline cartilage (arrow)

At least one ultrasound abnormality was found in almost all patients as detailed in Table 2. See Fig. 1 for ultrasound image examples of patients with HH-A. PD signals were more frequently detected in HH-A and HOA patients than in HH-WA patients, but still, half of patients in the latter group were PD-positive. GSS was more frequent in HOA than in HH-WA, whereas erosions and osteophytes occurred with a similar high frequency in all groups. GS and PD tenosynovitis were uncommon in all groups.

**Table 2:** Prevalence of ultrasound-detected pathologic changes

	HH-WA (*n* = 24)	HH-A (*n* = 26)	HOA (*n* = 38)
Erosions	10 (41.7)	12 (46.2)	20 (54.1)
Osteophytes	23 (95.8)	26 (100)	37 (100)
Grey scale synovitis	20 (83.3)	25 (96.2)	37 (100)**
Power Doppler synovitis	12 (50.0)	21 (80.8)*	30 (81.1)**
Grey scale tenosynovitis	4 (16.7)	7 (26.9)	9 (24.3)
Power Doppler tenosynovitis	0	2 (7.7)	4 (10.8)
Cartilage abnormalities	14 (58.3)	23 (88.5)* ^¥^	25 (67.6)
Calcium pyrophosphat dehydrate deposition	9 (37.5)	16 (61.5%)^¥¥^	4 (10.8)*

Data indicate number (percentage)

*HH-A* hereditary haemochromatosis with arthropathy, *HH-WA* hereditary haemochromatosis without arthropathy, *HOA* hand osteoarthritis

*p < 0.05 and **p = 0.01 compared to HH-WA, ^¥^ *p* = 0.05 and ^¥¥^ *p* < 0.001 compared to HOA; *p* values are not adjusted for multiple testing

Cartilage abnormalities were most common in patients with HH-A, which was mainly because of a high prevalence of cartilage damage in the MCP joints (HH-A, *n* = 22 (84.6%); HH-WA, *n* = 13 (54.2%); HOA, *n* = 21 (56.8%); *p* = 0.036). Similarly, CPPD deposition was most common in patients with HH-A as compared to patients with HH-WA or HOA.

**Fig. 2:**
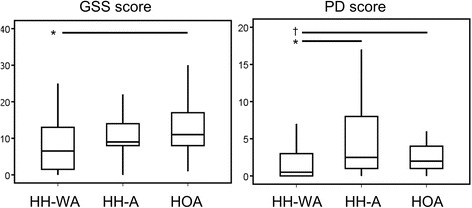
Ultrasound scores in patients with hereditary haemochromatosis and hand osteoarthritis. Total grey scale synovitis (GSS) and power Doppler (PD) scores are shown in patients with hereditary haemochromatosis without arthropathy (HH-WA), haemochromatosis with arthropathy (HH-A) and hand osteoarthritis (HOA). ^†^ *p* < 0.1 and *p < 0.05 for analysis using the Mann-Whitney *U* test (*p* values are not adjusted for multiple testing)

As depicted in Fig. 2, GSS (median score 9 (0–32) vs. 11.0 (1–30), respectively) and PD scores ([2.5 (0–17) vs. 2.0 (0–17), respectively) were similar in patients with HH-A and HOA but were higher in these groups as compared to patients with HH-WA (GSS 6.5 (0–25), *p* = 0.16 for comparison between HH-A and HH-WA and *p* = 0.026 between HOA and HH-WA; PD score 0.5 (0–9), *p* = 0.039 for comparison between HH-A and HH-WA and *p* = 0.097 between HOA and HH-WA). Erosion scores were similar in all groups (HH-A, 0 (0–7); HH-WA, 0 (0-5); HOA, 1 (0–7)), osteophyte scores were higher in HH-A (34 (4–75)) and HOA (36.0 (10–72)) than in HH-WA (23.5 (0–55); *p* = 0.024 for comparison between HH-A and HH-WA and *p* = 0.001 between HOA and HH-WA).

As detailed in Table 3, patients with HH-A and HH-WA had higher osteophyte, GSS and PD scores at the MCP joints than patients with HOA. Conversely, respective scores at the PIP and/or DIP joints were higher in HOA than in in HH.

**Table 3:** Scoring of ultrasound abnormalities according to joint region

	HH-WA	HH-A	HOA
Erosion score			
MCP	0 (0–5)	0 (0–7)	0 (0–7)
PIP	0 (0–1)	0 (0–2)	0 (0–4)
DIP	0	0	0 (0–4)
Osteophyte score			
MCP	4.5 (0–25)	12.5 (0–30)** ^¥¥^	5 (0–18)
PIP	5 (0–16) ^¥¥¥^	10 (0–28)^¥^	14 (2–30)
DIP	7.5 (0–13) ^¥¥¥^	8 (1–17)^¥¥¥^	16 (2–24)
Hip	2 (0–5) ^¥¥¥^	1 (0–6)^¥¥¥^	0 (0–4)
Knee	1.5 (0–5)	2 (0–6)	1 (0–6)
Grey scale synovitis score			
MCP	2 (0–12) ^¥¥^	3 (0–13)^¥¥¥^	1 (0–5)
PIP	1 (0–12) ^¥¥^	1.5 (0–12)	4 (0–16)
DIP	0 (0–5) ^¥¥¥^	1 (0–6)^¥¥¥^	5 (0–14)
Wrist	1 (0–4)	2 (0–4)* ^¥¥^	0 (0–4)
Hip	0 (0–1)	0 (0–2)	0 (0–1)
Knee	0 (0–2)	0 (0–5)	0 (0–2)
Ankle	0 (0–3)	0 (0–2)	0 (0–1)
Power Doppler synovitis score			
MCP	0 (0–5)	1.5 (0–11)** ^¥¥¥^	0 (0–2)
PIP	0 (0–3)	0 (0–6)	0 (0–9)
DIP	0 (0–2)	0 (0–1)	0 (0–5)
Wrist	0 (0–2)	1 (0–5)	0 (0–4)
Hip	0 (0–1)	0	0
Knee	0 (0–1)	0 (0–4)	0 (0–1)
Ankle	0 (0–2)	0 (0–3)	0
Grey scale tenosynovitis score			
MCP	0 (0–2)	0 (0–1)	0 (0–3)
PIP	0 (0–5)	0 (0–4)	0 (0–1)
DIP	0	0	0 (0–1)
Wrist	0 (0–2)	0 (0–1)	0 (0–1)
Ankle	0	0	0
Power Doppler tenosynovitis score			
MCP	0	0	0 (0–3)
PIP	0	0 (0–3)	0 (0–3)
DIP	0	0	0
Wrist	0	0	0 (0–2)
Ankle	0	0	0

Data indicate the median ultrasound score (range)

*HH-A* hereditary haemochromatosis with arthropathy, *HH-WA* hereditary haemochromatosis without arthropathy, *HOA* hand osteoarthritis, *MCP* metacarpophalangeal joints, *PIP* proximal interphalangeal joints, *DIP* distal interphalangeal joints

*p < 0.05 and **p ≤ 0.01 compared to HH-WA, ^¥^ *p* < 0.05, ^¥¥^ *p* ≤ 0.01 and ^¥¥¥^ *p* ≤ 0.001 compared to HOA; *p* values are not adjusted for multiple testing

Cartilage damage scores were highest in patients with HH-A (total score 11 (0–29)) as compared to patients with HH-WA (2.5 (0–25), *p* = 0.004) or HOA (2 (0–17), *p* < 0.001). Sub-analysis of scores of hands and knees revealed that the difference between groups was mainly related to cartilage damage at the MCP level (HH-A, 10.5 (0–27); HH-WA, 1.0 (0–24); HOA, 1.0 (0–15); *p* < 0.01 for comparison between HH-A and the other groups), whereas scores at the knees were comparable between groups (HH-A, 0.5 (0–5); HH-WA, 0 (0–6); HOA 0 (0–4)).

### Association between ultrasound findings and clinical parameters in patients with hereditary haemochromatosis associated arthropathy

In patients with HH-A, we observed moderate correlation between the osteophyte score and the number of bony swollen joints (correlation coefficient (corr_coeff_) 0.39, *p* = 0.05); however, neither of the ultrasound scores of inflammation or structural damage (nor their sub-scores for the hands) were linked with TJ, SJ, pain, global assessments or function.

A negative association between the global PD score and ferritin levels (corr_coeff_ −0.41, *p* = 0.039) was observed, and GSS and PD scores at the hands correlated with cartilage damage (corr_coeff_ 0.47, *p* = 0.001 and corr_coeff_ 0.6, *p* = 0.01, respectively).

### Association between ultrasound findings and x-ray scores in patients with hereditary haemochromatosis

The median global x-ray score was higher in HH-A (15.5 (1–48)) than in patients with HH-WA (5.5 (0–29), *p* = 0.007). Neither the global x-ray score nor the x-ray sub-score of hands was associated with pain, global assessments, function or ferritin levels.

In patients with HH-WA and HH-A, respectively, there was moderate to good correlation between the global x-ray score and the ultrasound osteophyte score (corr_coeff_ 0.70, *p* < 0.001 and corr_coeff_ 0.84, *p* < 0.001), GSS score (corr_coeff_ 0.40, *p* = 0.064 and corr_coeff_ 0.53, *p* = 0.006), PD score (corr_coeff_ 0.49, *p* = 0.019 and corr_coeff_ 0.70, *p* < 0.001) and cartilage score (corr_coeff_ 0.64, *p* = 0.001 and corr_coeff_ 0.83, *p* < 0.001).

### Reliability exercise

Inter-observer reliability of ultrasound findings was good, with ICCs of 0.94 (95% CI 0.69–0.99) for the osteophyte score, 0.78 (95% CI 0.14–0.96) for the GSS score, 0.73 (95% CI 0.1–0.95) for the PD score and 0.96 (95% CI 0.81–0.99) for the cartilage score. Reliability testing for erosions and tenosynovitis was not possible because of the small number of abnormal findings in the subset of patients who were tested.

## Discussion

In the present study, we demonstrate that ultrasound signs of inflammation and damage are common in patients with HH-A, particularly in the MCP joints. Albeit these findings had little impact on pain and function at the time of the clinical visit, they were associated with x-ray evidence of damage that in turn might lead to future disability [12, 23]. Patients without arthropathy also commonly had synovitis suggesting that inflammation might be present in HH despite the absence of, or clinically preceding, overt arthropathy.

Although arthropathy in patients with HH is common and joint symptoms are often disabling, systematic imaging studies investigating inflammatory and structural changes in HH-A have rarely been performed. A recent report highlighted the occurrence of PD synovitis in two cases of HH [24] and a small MRI study identified inflammatory and bony changes in the majority of patients with HH-A, and in up to 40% of patients without arthropathy [25]. Apart from the fact that MRI was only performed in the dominant hand, the definition of HH-A was unclear in that study. Nevertheless, these data support our observation of the inflammatory nature of HH-A, and they contrast the long-believed hypothesis that HH-A is purely a non-inflammatory disorder [26]. Given that signs of synovitis were present in the vast majority of patients with HH-A and HH-WA, it is tempting to speculate that these patients might benefit from anti-inflammatory treatment. Phlebotomies alone do not effectively reduce pain and have an unknown impact on the long-term progression of joint disease [27].

The overall extent of inflammatory and structural changes was similar in HH-A and HOA, an observation that fits with a previous histological study comparing synovial tissues in patients with rheumatoid arthritis (RA), HH-A and OA, who were undergoing joint replacement surgery [28]. In that study, the histological picture of HH-A resembled that of OA apart from a stronger neutrophilic infiltrate in HH-A samples, which was linked to increased haemosiderin deposition. In HH-A, inflammation and structural lesions were most prominent in the MCPs, whereas in HOA, GSS and osteophyte scores were higher in the PIPs and DIPs. This is consistent with the well-known predilection of HH and HOA, respectively for these anatomical sites [13, 27]. Median scores for erosions and tenosynovitis were zero for most joints, and apart from osteophytes, ultrasound abnormalities were uncommon in the hips, knees and ankles, suggesting that these lesions and joint regions have limited relevance for the ultrasound assessment of patients with HH.

One of the most intriguing findings of our study was the prominent cartilage damage in patients with HH-A, particularly in the MCP joints. In RA, a reduction in cartilage thickness has been shown to predict disability after 1–2 years, while it did not correlate with health assessment questionnaire (HAQ) scores at baseline [29, 30]. We also observed that neither ultrasound scores nor x-ray findings were associated with pain, global assessment and function; however, ultrasound abnormalities correlated strongly with x-ray-verified structural changes. A disparity between clinical and sonographic findings has also been reported for several other rheumatic diseases; however, ultrasound-verified inflammation consistently predicted future damage and functional restrictions in follow-up studies of RA and HOA [31–34]. Longitudinal studies are now also required in HH-A in order to clarify whether inflammation and cartilage abnormalities detected on ultrasound might predict future clinical and structural deterioration [23].

CPPD deposits were slightly more common in our cohort (37.5% in HH-WA and 61.5% in HH-A) than in previous cohorts of patients with HH (30–50%) [7]. We explain this by the greater sensitivity of ultrasound to detect CPPD as compared to other methods such as radiography or synovial fluid aspiration [35, 36].

The most important limitation of our study is the absence of a generally accepted definition of HH-A. While in some studies HH-A was defined as any joint pain associated with HH [25], we and others were more strict in accepting only joint pain plus characteristic radiographic changes to define HH-A [37, 38]. International classification criteria enabling specific differentiation of HH-A from mimicking conditions (e.g. HH with accompanying OA or RA) are urgently needed in order to conduct studies with comparable results [26].

Another limitation is that our patients with HH had long-standing disease and were on stable treatment, and that ferritin levels were thus within the normal range in almost all patients. Given that arthropathy in patients with HH has been linked with the duration and degree of iron overload [38, 39], it was impossible in our setting to investigate the direct contribution of iron load to the inflammatory burden.

Further, the ultrasound investigators were not blinded to the diagnoses of HH versus HOA (they were only blinded to the clinical findings). This might have influenced the results; however, given the similar results between HH-A and HOA in regard to several aspects (and assuming that a lack of blinding would have increased the difference between these groups) and the good inter-reader reliability between investigators, we believe that the bias due to lack of blinding to diagnosis was negligible.

## Conclusion

The prevalence of inflammatory and structural changes is high in patients with HH-A. Overall inflammation and osteophytes were comparable between patients with HH-A and HOA, whereas cartilage damage was more prominent in the former compared to the latter group. Scores for inflammation and damage were highest in the MCPs in patients with HH-A, whereas in HOA there were higher osteophyte and GSS scores for the PIPs and DIPs.

In patients with HH-WA, we observed a high prevalence of subclinical inflammation. These data demonstrate that HH-A is associated with synovial inflammation, contrasting with the long-believed hypothesis that joint pathology in HH is purely non-inflammatory.

## Abbreviations

corr_coeff_: Correlation coefficient
CPPD: Calcium pyrophosphate deposition
CRP: C-reactive protein
DIP: Distal interphalangeal joint
EGA: Evaluator’s global assessment of disease activity
ESR: Erythrocyte sedimentation rate
GS: Grey scale
GSS: Grey scale synovitis
GS-teno: Gey scale tenosynovitis
HAQ: Health assessment questionnaire
HH: Hereditary haemochromatosis
HH-A: Hereditary haemochromatosis with arthropathy
HH-WA: Hereditary haemochromatosis without arthropathy
HOA: Hand osteoarthritis
ICC: Intra-class correlation coefficient
JSN: Joint space narrowing
MCP: Metacarpophalangeal joint
MRI: Magnetic resonance imaging
NSAIDs: Non-steroidal anti-inflammatory drugs
OA: Osteoarthritis
PD: Power Doppler
PD-teno: Power Doppler signals related to tenosynovitis
PGA: Patients’ global assessment of disease activity
PIP: Proximal interphalangeal joint
RA: Rheumatoid arthritis
SJ: Swollen joints
TJ: Tender joints
VAS: Visual analogue scale
